# Symmetry breaking, Josephson oscillation and self-trapping in a self-bound three-dimensional quantum ball

**DOI:** 10.1038/s41598-017-16106-w

**Published:** 2017-11-22

**Authors:** S. K. Adhikari

**Affiliations:** Instituto de Física Teórica, UNESP - Universidade Estadual Paulista, 01.140-070 São Paulo, São Paulo, Brazil

## Abstract

We study spontaneous symmetry breaking (SSB), Josephson oscillation, and self-trapping in a stable, mobile, three-dimensional matter-wave spherical quantum ball self-bound by attractive two-body and repulsive three-body interactions. The SSB is realized by a parity-symmetric (a) one-dimensional (1D) double-well potential or (b) a 1D Gaussian potential, both along the z axis and no potential along the x and y axes. In the presence of each of these potentials, the symmetric ground state dynamically evolves into a doubly-degenerate SSB ground state. If the SSB ground state in the double well, predominantly located in the first well (z > 0), is given a small displacement, the quantum ball oscillates with a self-trapping in the first well. For a medium displacement one encounters an asymmetric Josephson oscillation. The asymmetric oscillation is a consequence of SSB. The study is performed by a variational and a numerical solution of a non-linear mean-field model with 1D parity-symmetric perturbations.

## Introduction

The topic of spontaneous symmetry breaking (SSB) in localized quantum states obeying Schrödinger dynamics has drawn much attention lately both in experimental^1^ and theoretical^2–7^ fronts. In simple two-boson non-relativistic quantum mechanics, parity is an exact symmetry and the ground state is non-degenerate. However, this symmetry can be broken even in non-relativistic quantum mechanics, involving many particles, bosons^2–4^ and fermions^8^. There have been numerous studies of SSB in non-linear optics using the non-linear Schrödinger (NLS) equation^9–11^. There have also been studies of SSB in a localized Bose-Einstein condensate (BEC)^12–14^. The simplest and commonly studied case of SSB in non-linear systems is an attractive one-dimensional (1D) BEC in a confining double-well potential with a doubly degenerate SSB ground state^2,3,5–7^. The SSB is found to appear in this system below a threshold of attractive non-linearity or above a threshold of the double-well barrier height^2^. Apart from the studies of SSB in a single-component BEC^3,4^, the other studies on this topic deal with trapped multi-component BEC^5–7^. The collapse instability of a two- (2D) or three-dimensional (3D) attractive BEC disappears in a 1D BEC^15^, hence the study of SSB in an attractive 1D BEC should be complimented by a similar study in a 2D and 3D BEC to verify if the SSB in an attractive 1D BEC does not take place exclusively in the domain of collapse in the 3D configuration. There has also been study of SSB in two-component trapped two-dimensional BEC where the symmetry breaking originating from a phase separation is achieved due to an interplay between inter-species interaction and trapping potential^16^.

The ground state of a *repulsive* 1D BEC in a double well is symmetric due to atomic repulsion with an equal number of atoms in both wells. If a small initial population imbalance between the two wells in created, repulsive atoms naturally move back and forth from one well to the another thus initiating a Josephson oscillation^17–20^. However, if the initial population imbalance is increased in a sufficiently repulsive BEC^21–24^ or a Fermi super-fluid^25^, a dynamical self-trapping of the atoms in one well takes place resulting in a net time-averaged population imbalance. The self-trapping of a larger number of atoms in one well compared to the other is counter-intuitive in a repulsive BEC.

Motivated by the above consideration, in this paper we study SSB, Josephson oscillation^17–20^ and self trapping in a 3D self-bound *attractive* matter-wave quantum ball^26–28^ placed in a parity-symmetric (a) 1D double-well potential or (b) a 1D Gaussian potential along the *z* axis. These 1D potentials are necessary for SSB; however, these potentials act in 1D and thus have no effect on the localization of the 3D quantum ball. The 3D quantum ball is stabilized in the presence of a repulsive three-body interaction and an adequate two-body attraction above a critical value^26–28^. It has been demonstrated that a tiny three-body repulsion stops the collapse and can stabilize a 3D quantum ball for zero^26^ and non-zero angular momenta^29^. This gives the unique opportunity to study SSB in a 3D quantum ball bound solely by non-linear interactions placed in these parity-symmetric 1D potentials. For the first time we detect an asymmetric Josephson oscillation because of the SSB in the quantum ball in the presence of these two 1D potentials. The quantum ball exhibits SSB in these potentials – the symmetric ground state is displaced from the origin along the *z* axis as a consequence of SSB, thus violating the parity symmetry of the Hamiltonian and leading to a doubly-degenerate ground state. The quantum ball can be displaced along either the positive or negative *z* axis leading to two degenerate states. We find that the SSB occurs for the weakest possible double-well or Gaussian potential.

The SSB takes place in an attractive BEC due to a sizable nonlinear interaction. Previous studies of SSB in trapped attractive BEC^2–4^ have employed a moderately large nonlinearity in a quasi-1D configuration. For such values of attractive nonlinearity, the corresponding trapped 3D BEC collapses, whereas the reduced 1D model does not collapse. In the present study, collapse has been stopped by a three-body repulsion and an adequate two-body attraction.

Unlike in a trapped repulsive BEC in a double-well potential with zero steady-state population imbalance, the population imbalance in a SSB ground state of a quantum ball in a double well is usually large. If the population imbalance in a SSB ground state, predominantly located in the first well (*z* > 0), is changed by displacing the ground state towards or away from the center of the double well (*z* = 0) by a small distance *δ*, a small oscillation of the quantum ball indicating self-trapping in the first well results. If the initial displacement is large and away from the trap center, one has a symmetric Josephson oscillation between the two wells. For a medium initial displacement towards or away from the trap center, an asymmetric Josephson oscillation between the two wells take place: two extreme states of the oscillating SSB BEC are not parity image of each other. This manifestly asymmetric Josephson oscillation is a consequence of SSB in the quantum ball in the presence of 1D double-well potential. For larger initial displacement towards the center of the trap, a self-trapping in the second well results. In the present case of the attractive quantum ball, the self trapping in the first and the second well for small and large initial displacements is not quite surprising, but the continued asymmetric Josephson oscillation of most atoms for a medium displacement is counter-intuitive as atomic attraction should permanently take all the atoms to one of the wells. The SSB also manifests when the self-bound quantum ball is placed on the top of a parity-symmetric 1D Gaussian potential hill. Like a classical ball the quantum ball is found to slide down the potential hill, thus spontaneously breaking the symmetry.

We base the present study on a variational approximation and a numerical solution of the 3D mean-field Gross-Pitaevskii (GP) equation in the presence of an attractive two-body and repulsive three-body interactions. The two-body contact attraction leads to a cubic non-linear term in the GP equation and an attractive cubic divergence near the origin in the effective Lagrangian, viz. Eq. (4), responsible for collapse instability. The three-body contact repulsion, on the other hand, leads to a quintic non-linearity and a repulsive sextic divergence near the origin suppressing the attractive cubic divergence, thus stopping the collapse.

The mathematical structure of the non-linear mean-field GP equation is the same as that of the NLS equation used to study pulse propagation in non-linear optics, although the physical meaning of the different terms is distinct in two cases. Hence, in a cubic-quintic non-linear medium^30–32^ one can have a stable mobile 3D spatiotemporal light bullet^28,29^ and a SSB can occur in that context also.

We present the 3D GP equation used in this study in Sec. 2.1 and an analytic variational approximation to it. In Sec. 2.2 we present the numerical and variational results for stationary profiles of SSB quantum ball under perturbations in the form of a 1D double-well or a 1D Gaussian potential. We study how a parity-symmetric state dynamically evolves into a SSB state under the action of the perturbation. We study Josephson oscillation and self-trapping of the SSB quantum ball in the 1D double-well potential. A description of the numerical methods for the solution of the GP equation is given in Sec. 3. We end with a summary and discussion in Sec. 4.

## Result

### The GP equation and Variational approximation

The mean-field GP equation describing the BEC quantum ball in the presence of an attractive two-body and a repulsive three-body interaction subject to an external perturbation *V*(*z*) is given by^26,27^

1 $$i\hslash \frac{{\rm{\partial }}\varphi ({\bf{r}},t)}{{\rm{\partial }}t}=[-\frac{{\hslash }^{2}}{2m}{{\rm{\nabla }}}_{{\bf{r}}}^{2}+V(z)-\frac{4\pi |a|{\hslash }^{2}N}{m}|\varphi ({\bf{r}},t{)|}^{2}+\frac{\hslash {N}^{2}{K}_{3}}{2}|\varphi ({\bf{r}},t{)|}^{4}]\varphi ({\bf{r}},t),\quad \int |\varphi ({\bf{r}},t){|}^{2}d{\bf{r}}=1,$$

2 $$V(z)=\frac{{c}_{1}}{2}m{\omega }^{2}{z}^{2}+{c}_{2}\hslash \omega {e}^{-\gamma m\omega {z}^{2}/\hslash },$$

where *m* is the mass of each atom, *ϕ*(**r**,*t*) is the condensate wave function at a space point ${\bf{r}}=\{x,\,y,\,z\}$ and time *t*, *a* is the *s*-wave scattering length of atoms taken here to be negative (attractive), *K*_3_ is the three-body interaction term, and *N* is the number of atoms. The external double-well potential *V*(*z*) consists of a harmonic potential of strength *c*_1_ and angular frequency *ω* and a Gaussian potential of strength *c*_2_ and width parameter *γ*. If *c*_1_ is set zero, this potential becomes a Gaussian potential. Equation (1) can be written in the following dimensionless form after a redefinition of the variables

3 $$i\frac{{\rm{\partial }}\varphi ({\bf{r}},t)}{{\rm{\partial }}t}=[-\frac{{{\rm{\nabla }}}_{r}^{2}}{2}+\frac{{c}_{1}}{2}{z}^{2}+{c}_{2}{e}^{-\gamma {z}^{2}}-p|\varphi ({\bf{r}},t{)|}^{2}+q|\varphi ({\bf{r}},t{)|}^{4}]\varphi ({\bf{r}},t),$$

where $p=4\pi Na/l$, $q=m{N}^{2}{K}_{3}/(2\hslash {l}^{4})$, length is scaled in units of $l\equiv \sqrt{\hslash /m\omega }$, time in units of $m{l}^{2}/\hslash$, $|{\varphi }{|}^{2}$ in units of *l* ^−3^, and energy in units of $\hslash \omega$. For a stationary state with property ${\varphi }({\bf{r}},t)\sim {\varphi }({\bf{r}})\exp (-i\mu t)$, with *μ* the chemical potential, one has the following time-independent GP equation:

4 $$\mu \varphi ({\bf{r}})=[-\frac{{{\rm{\nabla }}}_{r}^{2}}{2}+\frac{{c}_{1}}{2}{z}^{2}+{c}_{2}{e}^{-\gamma {z}^{2}}-p|\varphi ({\bf{r}}{)|}^{2}+q|\varphi ({\bf{r}}{)|}^{4}]\varphi ({\bf{r}}).$$

For an analytic understanding of SSB of a quantum ball, we consider the Lagrange variational formulation^33^ to Eq. (4). In this axially symmetric problem, convenient analytic Gaussian variational approximation of the quantum ball wave function is^33^

5 $$\varphi ({\bf{r}})=\frac{{\pi }^{-3/4}}{{\sigma }_{1}{\sigma }_{2}^{1/2}}\exp [-\frac{{\rho }^{2}}{2{\sigma }_{1}^{2}}-\frac{{(z-{z}_{0})}^{2}}{2{\sigma }_{2}^{2}}],$$

where $\rho =\sqrt{{x}^{2}+{y}^{2}},$ σ_1_and σ_2_ are radial and axial widths, respectively. In this approximation, the Gaussian in the *z* direction is displaced by a distance *z*_0_ from the origin due to the SSB under perturbation *V*(*z*). The *z*-profile of the displaced SSB quantum ball is not strictly a Gaussian, but is close to it, as we will see, and here for simplicity we take it as a Gaussian. The (generalized) Lagrangian density corresponding to Eq. (4) is

6 $${ {\mathcal L} }({\bf{r}})=\frac{|\nabla {\varphi }({\bf{r}}{)|}^{2}}{2}+\frac{{c}_{1}}{2}{z}^{2}|{\varphi }({\bf{r}}{)|}^{2}+{c}_{2}{e}^{-\gamma {z}^{2}}|{\varphi }({\bf{r}}{)|}^{2}-\frac{p}{2}|{\varphi }({\bf{r}}{)|}^{4}+\frac{q}{3}|{\varphi }({\bf{r}}{)|}^{6}\mathrm{.}$$

Equation (4) can be obtained by extremizing the functional (6)^33^. Consequently, the effective Lagrangian functional $\overline{L}({\sigma }_{1},{\sigma }_{2},{z}_{0})\equiv 2\pi \int { {\mathcal L} }({\bf{r}})dz\rho d\rho$ becomes

7 $$\overline{L}=\frac{1}{2{\sigma }_{1}^{2}}+\frac{1}{4{\sigma }_{2}^{2}}+\frac{{c}_{1}}{4}({\sigma }_{2}^{2}+2{z}_{0}^{2})+\frac{{c}_{2}{e}^{-\frac{\gamma {z}_{0}^{2}}{1+\gamma {\sigma }_{2}^{2}}}}{\sqrt{1+\gamma {\sigma }_{2}^{2}}}-\frac{p{\pi }^{-\mathrm{3/2}}}{4\sqrt{2}{\sigma }_{1}^{2}{\sigma }_{2}}+\frac{q{\pi }^{-3}}{9\sqrt{3}{\sigma }_{1}^{4}{\sigma }_{2}^{2}}\mathrm{.}$$

Lagrangian (7) is the energy per atom in the SSB quantum ball. The variational parameters $\nu \equiv {\sigma }_{1},{\sigma }_{2},{z}_{0}$ are obtained from a minimization of the effective Lagrangian functional $\overline{L}:$

8 $$\frac{\partial \overline{L}}{\partial \nu }=0.$$

### Numerical Results

For illustration in this paper, we consider attractive ^7^Li atoms with scattering length $a=-27.4{a}_{0}$ ^34^ and a variable three-body interaction term *K*_3_, where *a*_0_ is the Bohr radius. The three-body atom loss rate due to the formation of molecules is not accurately known^35^ for relatively low-density quantum balls in the trap-less domain employed in this study. The effect of this loss rate is expected to be considerable in a large high-density trapped attractive BEC and will be negligible for the small-time dynamics (of about 5 ms) of untrapped quantum balls presented in this paper, as was demonstrated in ref.^26^, and hence is not considered here. We take the harmonic oscillator length *l* = 1 *μ*m, which corresponds to a trap of angular frequency $\omega =2\pi \times 1444$ s^−1^, unit of time ${t}_{0}\equiv m{l}^{2}/\hslash =0.11$ ms, and of energy $\hslash \omega =9.57\times {10}^{-31}$ J. The parameters of the double-well potential (2) are taken as *c*_1_ = *c*_2_ = 1 and *γ* = 20 and of the Gaussian potential as *c*_1_ = 0, *c*_2_ = 1 and *γ* = 20.

A stable quantum ball corresponds to a global minimum of the conserved effective Lagrangian $\overline{L}({\sigma }_{1},{\sigma }_{2},{z}_{0})$ (7). At the center of the σ_1_-σ_2_ plane, ${\sigma }_{1},{\sigma }_{2}\to 0$, and the Lagrangian $\overline{L}({\sigma }_{1},{\sigma }_{2},{z}_{0})\to +\infty$, which guarantees the absence of a collapsed state at the origin. The statics and the dynamics of a self-bound 3D quantum ball in the absence of the 1D double well (*c*_1_ = *c*_2_ = 0) have been studied in details^26,27^. The quantum ball is bound for any value of the quintic non-linearity *q* and for the cubic non-linearity *p* above a critical value *p* > *p*_crit_^26,27^.

**Figure 1:**
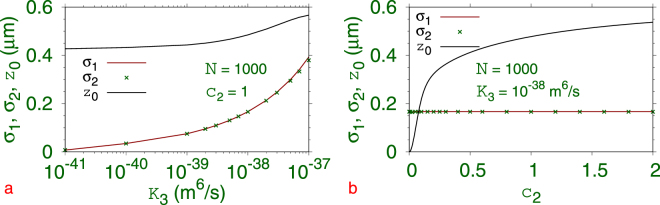
Variational parameters ${\sigma }_{1},{\sigma }_{2},{z}_{0}$, from a minimization of the Lagrangian (7), (**a**) versus *K*_3_ for *c*_2_ = 1 and (**b**) versus *c*_2_ for ${K}_{3}={10}^{-38}$ m^6^/s. The other parameters are $N=\mathrm{1000,}\,a=-27.4{a}_{0},\,{c}_{1}=\mathrm{1,}\,\gamma =20.$

We study the SSB states in the 1D double-well potential (2) from a minimization of the variational Lagrangian (7). This determines the variational widths σ_1_,σ_2_ and the parameter *z*_0_ which is a measure of symmetry breaking. We consider $N=1000$ ^7^Li atoms. In Fig. 1(a) we plot the variational parameters ${\sigma }_{1},{\sigma }_{2}$ and *z*_0_ versus the three-body interaction term *K*_3_ for parameters *c*_1_ = *c*_2_ = 1, *γ* = 20 in Eq. (2). In Fig. 1(b) we show the same parameters versus *c*_2_ for three-body interaction ${K}_{3}={10}^{-38}$ m^6^/s and *c*_1_ = 1. We find that for any non-zero *c*_2_, *z*_0_ is non zero: a non-zero *c*_2_ with *c*_1_ = 1 in Eq. (2) represents a double well and a non-zero *z*_0_ signals SSB. Hence, a symmetry breaking will take place for the weakest possible 1D double-well potential.

To study the density distribution of the quantum balls, we define reduced 1D densities by

9 $${\rho }_{{\rm{1D}}}(x)=\int dzdy|{\varphi }({\bf{r}}{)|}^{2},$$

10 $${\rho }_{{\rm{1D}}}(z)=\int dxdy|{\varphi }({\bf{r}}{)|}^{2}\mathrm{.}$$

**Figure 2:**
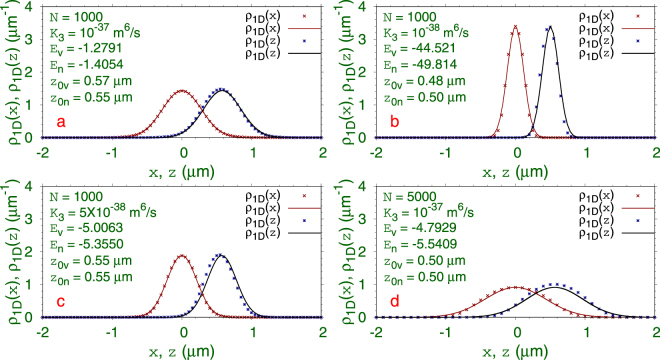
Numerical (points) and variational (line) 1D reduced densities ${\rho }_{1D}(x)$ and ${\rho }_{1D}(z)$ of SSB quantum balls of $N=1000$ ^7^Li atoms and different three-body term *K*_3_ in the double-well trap (2) with *c*_1_ = *c*_2_ = 1, *γ* = 20. The exhibited numerical (n) and variational (v) energies per atom given by Eq. (7) are in units of 9.57 × 10^−31^ J. The peak of the density ${\rho }_{1D}(z)$ at *z*_0_ and its asymmetric distribution is reasonably well represented by the symmetric Gaussian form of the variational approximation.

The 3D numerical simulation of the GP equation is much more complicated and time consuming compared to the variational approximation considered above. However, to validate the variational findings, for example in Fig. 1, a comparison to actual numerical results is called for, which we undertake next. The result of this investigation is illustrated in Fig. 2, where we compare the reduced 1D densities for several SSB bound states along the *x* and *z* axes as obtained from variational approximation and numerical solution of the GP equation with the double well (2) with parameters *c*_1_ = *c*_2_ = 1, *γ* = 20. The variational and numerical energies per atom, as given by Lagrangian (7), are also shown in the respective plots. For a fixed number of atoms, $N=1000$, the quantum ball is smaller in size (compact) for a small three-body term *K*_3_. For a fixed three-body term *K*_3_, the quantum ball is more compact for a small number of atoms. The SSB is also explicit in Fig. 2: the reduced density along *z* direction is asymmetric and does not have any symmetry around *z* = 0 or around *z* = *z*_0_ – the point of density maximum. The SSB quantum balls of Fig. 2 are created in one of the doubly-degenerate states centered at *z* = *z*_0_, the other degenerate state is located at *z* = −*z*_0_. The wave functions of these two degenerate states are *z*-parity images of each other: ${{\varphi }}_{1}(x,y,z)={{\varphi }}_{2}(x,y,-z)$. Considering that the reduced 1D density ${\rho }_{1D}(z)$ is not symmetric around *z* = *z*_0_ implying a non-Gaussian *z* profile of the quantum ball, the agreement between the variational and numerical densities is good.

**Figure 3:**
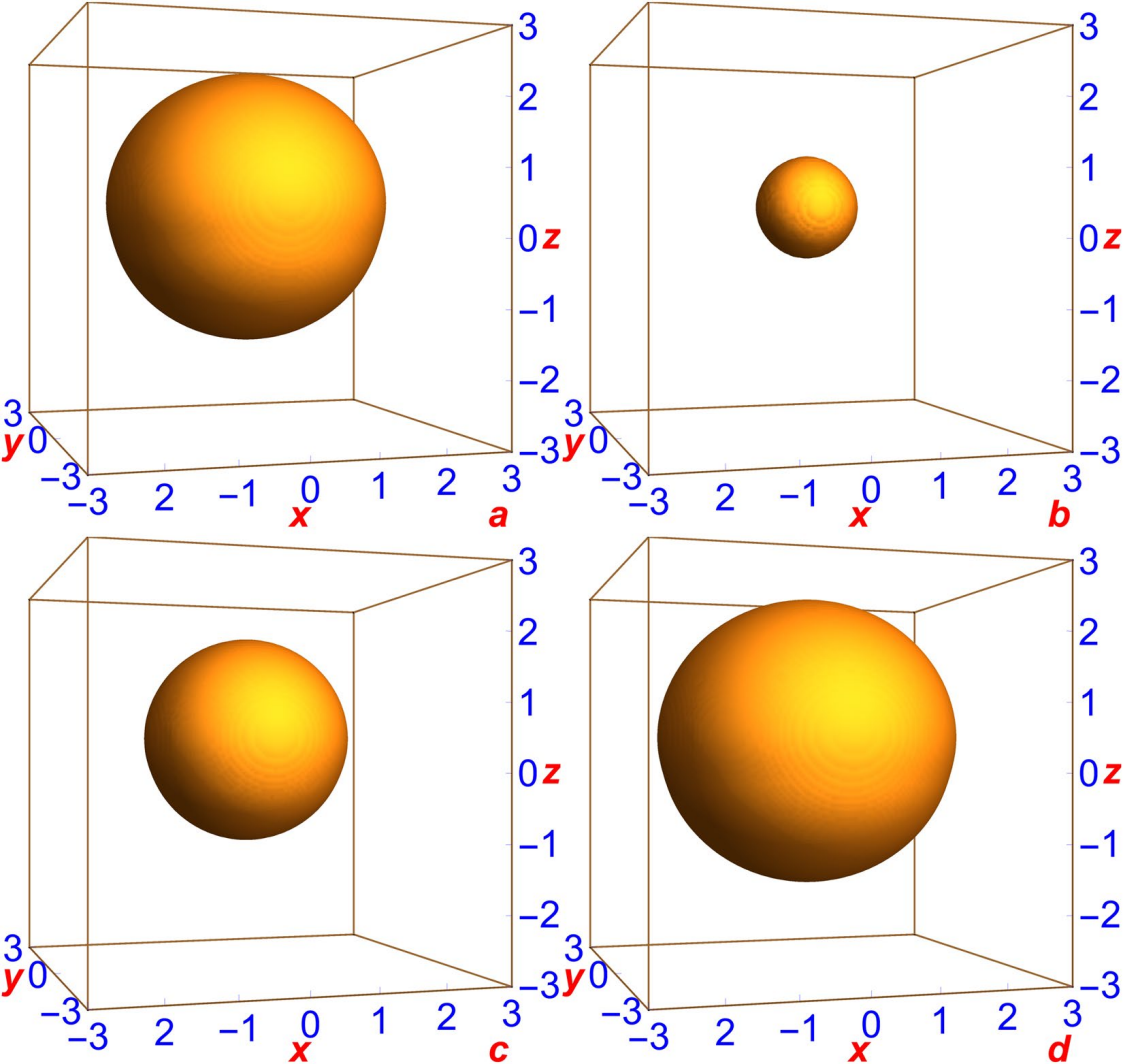
Three-dimensional isodensity contour of SSB quantum balls ($N|{\varphi }({\bf{r}}{)|}^{2}$) in 1D double-well potential (2) with *c*_1_ = *c*_2_ = 1, *γ* = 20. The parameters in (**a**)–(**d**) are the same as in Fig. 2(a)–(d), respectively: (**a**) $N=\mathrm{1000,}\,{K}_{3}={10}^{-37}$ m^6^/s, (**b**) $N=\mathrm{1000,}\,{K}_{3}=\times {10}^{-38}$ m^6^/s, (**c**) $N=\mathrm{1000,}\,{K}_{3}=5\times {10}^{-38}$ m^6^/s, (**d**) $N\mathrm{=5000,}\,{K}_{3}=3\times {10}^{-37}$ m^6^/s. The density of atoms on the contour is 10^9^ atoms/cm^3^. The units of $x,y,z$ are *μ*m.

We now consider the 3D profile of the SSB quantum ball in the 1D double-well potential. In Fig. 3(a)–(d), we plot the 3D isodensity contour ($N|{\varphi }({\bf{r}}{)|}^{2}$) of the quantum balls exhibited in Fig. 2(a)–(d), respectively. In Fig. 3 we see that as a result of SSB the ball is physically displaced along the *z* axis slightly in the positive *z* direction, which is also implicit in Fig. 2. The displaced quantum ball is not spherical, but slightly deformed in the *z* direction. The free quantum ball in the absence of the double-well potential is spherical.

**Figure 4:**
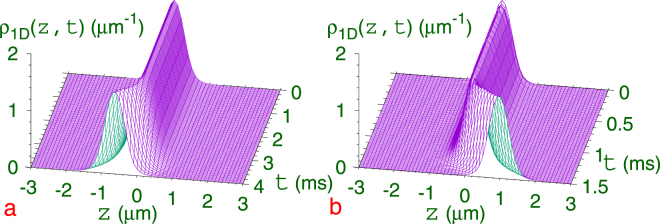
Spontaneous symmetry breaking of a quantum ball made of $N=1000$ ^7^Li atoms with $a=-27.4{a}_{0}$ and ${K}_{3}={10}^{-37}$ m^6^/s by real-rime simulation through a plot of 1D density ${\rho }_{1D}(z,t)$ versus *z* and *t*. (**a**) A self-bound quantum ball is initially placed at the top of a hill potential ${c}_{2}{e}^{-\gamma {z}^{2}}$ (c_2_ = 0.05, *γ* = 20) to study the SSB dynamics. (**b**) A quantum ball created in the harmonic well ${z}^{2}/2$ is placed at the top of the hill at *r* = 0 of the double well ${z}^{2}\mathrm{/2}+{c}_{2}{e}^{-\gamma {z}^{2}}$ (*c*_2_ = 0.4, γ = 20) for the SSB dynamics.

To demonstrate the dynamical transition of a quantum ball from a parity-symmetric to a SSB state, we consider a Gaussian hill potential ${c}_{2}{e}^{-\gamma {z}^{2}}$ with *c*_1_ = 0 in Eq. (3). Like a classical ball, the quantum ball placed at the top of a hill will slide down the hill spontaneously breaking the symmetry. We consider a self-bound quantum ball with $N=1000$ ^7^Li atoms with ${K}_{3}={10}^{-37}$ m^6^/s as obtained by solving Eq. (4) with *c*_1_ = *c*_2_ = 0 by imaginary-time propagation. The quantum ball so obtained is placed at the top of the hill *r* = 0 and the dynamics studied by solving Eq. (4) with *c*_1_ = 0, *c*_2_ = 0.05, *γ* = 20. The quantum ball stays at the top of the hill in unstable equilibrium for some time, but eventually slides down the hill away from the position of unstable equilibrium spontaneously breaking the symmetry. The interval of time for SSB to start is large when the height of the hill *c*_2_ is small and its width large and vice versa. This is next demonstrated by real-time simulation for a small *c*_2_ = 0.05. The SSB is illustrated in Fig. 4(a), where we plot the 1D density ${\rho }_{{\rm{1D}}}(z,t)$ versus *z* and *t* during this dynamics. At about $t\approx 3$ ms, the 1D density of the quantum ball is deviated from the central position indicating the motion of the ball down the hill. Once the motion is started the ball will move away from the hill reducing the energy of the system.

Although, a simple hill potential demonstrates SSB, it is not very convenient for studying a controlled dynamics in a confined space. To study the dynamics of a SSB state of the quantum ball in a controlled way, we consider next the double-well potential of Eq. (4): c1 = 1, c_2_ ≠ 0. First, by imaginary-time simulation we calculate the ground state of the quantum ball with $N=1000$ and ${K}_{3}={10}^{-37}$ m^6^/s in the single harmonic well: *c*_1_ = 1, *c*_2_ = 0. The quantum ball so obtained is placed at the top of the hill *r* = 0 in the double-well potential and the dynamics studied by solving Eq. (4) with *c*_1_ = 1, *c*_2_ = 0.4, *γ* = 20. The SSB dynamics is illustrated in Fig. 4 (b), where we again plot the 1D density ${\rho }_{{\rm{1D}}}(z,t)$ versus *z* and *t*. At about $t\approx 1$ ms the SSB is manifested and the quantum ball slides down the hill of the double well. However, different from Fig. 4(a), after sliding down the hill the quantum ball now remains confined in space due to the infinite harmonic trap of the double well. In both cases – Fig. 4(a) and (b) – there is no preferred direction of sliding from the top of the hill; it is decided by the development of numeric in real-time simulation.

Usual Josephson oscillation in cold atoms considers a repulsive trapped BEC through a narrow barrier. Different from that scenario, next we study the Josephson oscillation of the attractive 3D quantum ball through the narrow barrier of the 1D double-well potential in the *z* direction with no trap in the perpendicular directions *x* and *y*. To this end, we first solve the GP equation by imaginary-time propagation to obtain the doubly-degenerate SSB stationary state of the quantum ball in the presence of the double-well potential along the *z* axis, which we use subsequently in the study of Josephson oscillation. This SSB stationary state is displaced from the center at *z* = 0. The Josephson oscillation is started by giving the pre-calculated SSB stationary state in the double-well potential a displacement *δ* along the positive *z* axis. A positive *δ* denotes a displacement along the positive *z* axis away from the center of the trap, whereas a negative *δ* denotes a displacement along the negative *z* axis towards the center of the trap. The resultant dynamics is studied by real-time simulation of the GP equation (3) with the displaced quantum ball in the initial state. To quantify the Josephson oscillation we consider the dynamics of population imbalance *S*(*t*)

11 $$S(t)\equiv \frac{{N}_{1}(t)-{N}_{2}(t)}{{N}_{1}(t)+{N}_{2}(t)},$$

where ${N}_{1}(t)$ and ${N}_{2}(t)$ are the number of atoms at time *t* for *z* > 0 and *z* < 0, respectively.

**Figure 5:**
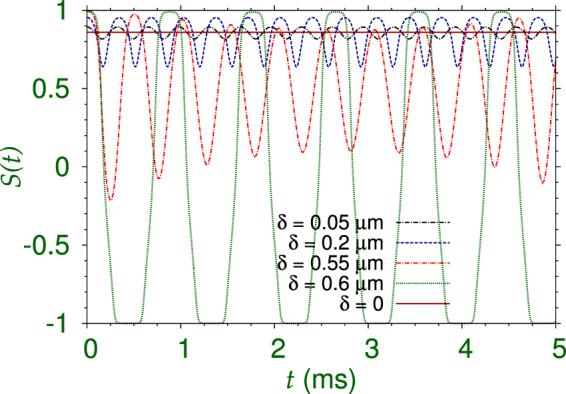
Josephson oscillation and self-trapping of the SSB quantum ball of Fig. 2(c) ($N=\mathrm{1000,}\,{K}_{3}=5\times {10}^{-38}$ m^6^/s) from a dynamics of population imbalance between the two wells. The dynamics is obtained by a real-time evolution of the GP equation (3) after giving an initial displacement *δ* along the positive *z* axis to the SSB stationary state. The positive *δ* values indicate a displacement of the initial state away from the trap center at *z* = 0. For smaller initial displacements, ($\delta =+0.05$ *μ*m, $+0.2$ *μ*m) the quantum ball is predominantly in the *z* > 0 domain denoted by an average positive $S(t)$ indicating self-trapping in the first well, whereas for larger displacements $(\delta =+0.55$ *μ*m) a Josephson oscillation is initiated with a tunneling of most atoms between the two wells. For larger displacements ($\delta \ge +0.6$ *μ*m) a symmetric Josephson oscillation takes place with a tunneling of all atoms between the two wells.

For an illustration of Josephson oscillation and self trapping, we consider the SSB quantum ball shown in Fig. 2(c) with $N=\mathrm{1000,}\,{K}_{3}=5\times {10}^{-38}$ m^6^/s. The Josephson oscillation dynamics in this case is illustrated first for positive *δ* (initial displacement away from the trap center *z* = 0) in Fig. 5 through plots of *S*(*t*) versus *t* for $\delta =+0.6$ *μ*m, + 0.55 *μ*m, + 0.2 *μ*m, + 0.05 *μ*m. The initial SSB of Fig. 2(c) with $S\mathrm{(0)}=0.86$ has 93% of atoms in the first well with *z* > 0. For smaller displacements, $\delta =+0.05$ *μ*m and + 0.2 *μ*m, the oscillation of *S*(*t*) for solely positive values in Fig. 5 indicates self-trapping of most of the atoms in the first well: *z* > 0. However, for larger initial displacement, e.g., $\delta =+0.55$ *μ*m, the oscillation of *S*(*t*) covering both positive and negative values indicates Josephson oscillation with most of the atoms oscillating between the two wells. In this case *S*(*t*) oscillates between the limiting values +0.98 and −0.3 indicating that the percentage of atoms in the second well, *z* < 0, varies between 1% and 65%. This is a case of asymmetric Josephson oscillation, the asymmetry is introduced due to the SSB of the initial state located in the first well: *z* > 0. In the case of Josephson oscillation in a trapped repulsive BEC, the initial state is parity symmetric and so is the Josephson oscillation^21–24^. For a slightly larger initial displacement, e.g., $\delta \ge +0.6$ *μ*m, *S*(*t*) oscillates between the limits $\pm 1$ indicating a transfer of all atoms from one well to another and *vice versa* during a symmetric Josephson oscillation.

**Figure 6:**
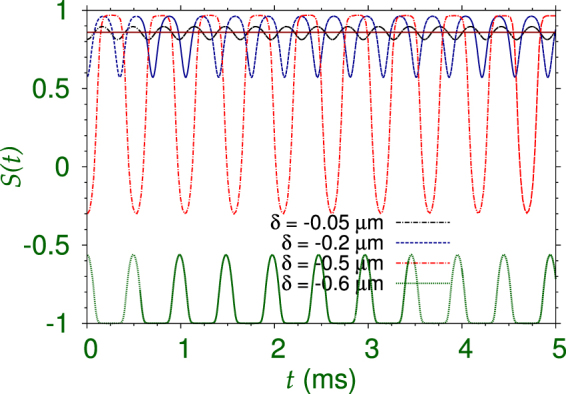
Josephson oscillation and self-trapping of the SSB quantum ball of Fig. 2(c) from a dynamics of population imbalance between the two wells. Refer to Fig. 5 and the text for a complete description. The negative *δ* values indicate a displacement of the initial state towards the the trap center at *z* = 0. For smaller initial displacements, ($|\delta |=+0.05$ *μ*m, +0.2 *μ*m) the quantum ball is predominantly in the *z* > 0 domain denoted by an average positive *S*(*t*) indicating self-trapping in the first well, whereas for larger displacements $(|\delta |=+0.5$ *μ*m) a Josephson oscillation is initiated with a tunneling of most atoms between the two wells. For larger displacements ($|\delta |=+0.55$ *μ*m), a permanent self trapping in the second well takes place indicated by negative *S*(*t*) vales.

The Josephson oscillation dynamics for negative *δ* values (displacement towards the center of the trap at *z* = 0) of the SSB quantum ball of Fig. 2(c) is studied next. For small values of initial displacement, $|\delta |=0.05$ *μ*m, 0.2 *μ*m, the population imbalance *S*(*t*) is always positive indicating a self trapping of most atoms in the first well: *z* > 0. However, for a medium initial displacement, $|\delta |=0.5$ *μ*m, a significant percentage of atoms could be transferred to the second well during Josephson oscillation dynamics, while *S*(*t*) oscillates between positive and negative values +0.97 and −0.3, indicating that the percentage of atoms in the second well, *z* < 0, varies between 2% and 65%. For a larger initial displacement $|\delta |=0.6$ *μ*m, most atoms of the quantum ball enters the second well (*z* < 0) at *t* = 0 with $S\mathrm{(0)}\approx -0.55$ and the quantum ball is accommodated in the position of the second of the doubly-degenerate SSB states in the second well and oscillates around this position leading to negative *S*(*t*) values indicating a permanent self-trapping in the second well. We find that the dynamics for $\delta =+0.05$ *μ*m, +0.2 *μ*m and +0.55 *μ*m is surprisingly similar to that for $\delta =-0.05$ *μ*m, −0.2 *μ*m and −0.5 *μ*m, respectively. For small displacements away from the center, viz. Figure 5, or towards the center, viz. Figure 6, the quantum ball oscillates around the position of equilibrium, thus leading to similar dynamics in Figs 5 and 6. However, larger displacements towards the center of the trap takes the quantum ball in the initial state close to the position of equilibrium of the second degenerate state at z < 0 and the quantum ball starts oscillating around this position, viz. $\delta =-0.6$ *μ*m in Fig. 6. For a larger initial displacement away from the trap, viz. $\delta =+0.6$ *μ*m in Fig. 5, the quantum ball reaches the position of equilibrium of the second degenerate state at *z* < 0 with a large kinetic energy. Consequently, it cannot be trapped in this position and executes a symmetric Josephson oscillation through the Gaussian barrier at the center of the double well. Even larger initial displacement towards the center of the trap takes the quantum ball deep into the second well past the position of equilibrium of the second degenerate state at *z* < 0 and essentially the same dynamics as illustrated in Figs 5 and 6 emerges, however, with *S*(*t*) changed to −*S*(*t*) due to the *z*-parity symmetry of the problem.

The reduced 1D density in the *z* direction ${\rho }_{1D}(z)$ of the SSB quantum-ball is not symmetric around the *z* peak at *z* = *z*_0_. This is already explicit in Fig. 6. The *z* peak of the SSB quantum ball of Fig. 2(c) is located at $z\equiv {z}_{0}\approx 0.55$. If we give a displacement of this quantum ball through $\delta =-0.5$ *μ*m and $\delta =-0.6$ *μ*m towards center, respectively, the quantum ball will come to the symmetric positions $z\approx \pm 0.05$ *μ*m. As the Hamiltonian is *z*-parity symmetric, these two *z*-symmetric initial configurations, should lead to similar oscillation dynamics (one being parity image of other), if the density ${\rho }_{1D}(z)$ were symmetric around the peak at *z* = *z*_0_. However, the vastly different asymmetric nature of oscillation dynamics for $\delta =-0.5$ *μ*m and $\delta =-0.6$ *μ*m in Fig. 6 is due to the asymmetric nature of the peak in density ${\rho }_{1D}(z)$.

**Figure 7:**
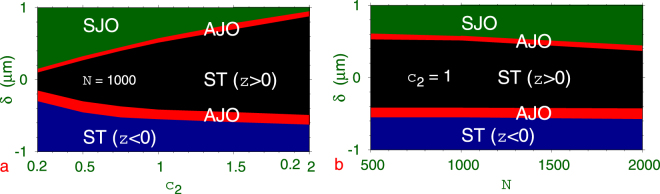
Phase plot in the (**a**) $\delta -{c}_{2}$ plane ($N=1000$) and (**b**) $\delta -N$ plane (*c*_2_ = 1) illustrating the symmetric (SJO, Green) and asymmetric (AJO, Red) Josephson oscillation, and self-trapping (ST) for *z* > 0 (Black) and *z* < 0 (Blue) of a 3D quantum ball in a double-well potential along the *z* axis. The fixed parameters of the quantum ball and the double-well potential are ${K}_{3}=5\times {10}^{-38}$ m^6^/s, $a=-27.4{a}_{0},$ *c*_1_ = 1, $\gamma =20$.

Next we illustrate in Fig. 7 the appearance of the symmetric (SJO) and asymmetric (AJO) Josephson oscillations, and self-trapping ST for *z* > 0 and *z* < 0 of a 3D quantum ball in a double-well potential along the *z* axis in a phase-plot in the (a) $\delta -{c}_{2}$ plane and (b) $\delta -N$ plane. The initial SSB quantum ball in the double-well potential is located in *z* > 0. These plots are not symmetric around $\delta =0$. For large positive *δ* (displacement away from *z* = 0) we have symmetric Josephson oscillation. However, for large negative *δ* (displacement towards *z* = 0) we have self-trapping in the *z* < 0 domain. For small $|\delta |$ we have self trapping in the *z* > 0 domain. The transition from one regime to another is sharp, e.g., for a small change in the model parameter *δ*, one can have transition from a symmetric Josephson oscillation to an asymmetric Josephson oscillation, then to self-trapping, etc. The self-trapping corresponds to a disbalance of time-averaged atom population in the two wells of the double-well potential or a non-zero time-averaged $\langle S(t)\rangle$ of Eq. (11): $\langle S(t)\rangle =0$ for the parameter domain of symmetric Josephson oscillation and $\langle S(t)\rangle \ne 0$ for all other domains illustrated in Fig. 7, viz. Figures 5 and 6.

## Methods

The 3D GP equation (3) is generally solved by the split-step Crank-Nicolson^36–38,46^ and Fourier spectral^39^ methods. The split-step Crank-Nicolson method in Cartesian coordinates is employed in the present study. We use a space ($r$ $=\{x,y,z\}$) step of 0.05*μ*m∼0.025 *μ*m, a time step of 0.0005*t*_0_ ms∼0.00025*t*_0_ ms^36–38^ and the number of discretization points 192∼256 in each of *x*,*y* and *z* directions. There are different C and FORTRAN programs for solving the GP equations^36,37,40–43^ and one should use an appropriate one. We use both imaginary- and real-time propagation^36–38^ for the numerical solution of the 3D GP equation. The imaginary-time propagation is used to find the stationary state and the real-time propagation is used in the study of dynamics employing the initial stationary profile obtained by the imaginary-time propagation. In the imaginary-time propagation the initial state was taken as in Eq. (5) with the parameters obtained from the variational solution (8).

## Discussion

To summarize, we demonstrate the formation of SSB (spontaneous symmetry-broken) doubly-degenerate states of a 3D quantum ball bound by attractive two-body and repulsive three-body interactions in a 1D double-well and in a Gaussian potential along the *z* direction employing a variational approximation and a numerical solution of the 3D GP equation. The doubly-degenerate states are symmetrically located at $z=\pm {z}_{0}$. The double-well and Gaussian potentials have no effect on the binding of the quantum ball but are required for symmetry breaking. We also study Josephson oscillation and self-trapping of the 3D quantum ball in the 1D double-well potential along the *z* axis. An oscillation of a quantum ball in the first well (*z* > 0) is initiated by giving a displacement to it in the *z* direction and the subsequent dynamics is studied to investigate Josephson oscillation and self-trapping. For a small initial displacement towards or away from the center of the trap (*z* = 0), one has oscillation of the quantum ball mostly in the first well indicating self-trapping in the first well. For a large initial displacement away from the center, one encounters symmetric Josephson oscillation of the quantum ball between the two wells. For a similar displacement towards the center, a self-trapping of the quantum ball in the second well (*z* < 0) is realized. For a medium displacement towards the center or away from the center, one has an asymmetric Josephson oscillation of the quantum ball between the two wells. The relatively large asymmetry of the Josephson oscillation is due to asymmetric profile of the quantum ball on two sides of the density peak in the *z* direction at *z* = *z*_0_ and is an earmark of spontaneous symmetry breaking.

In this paper we considered a self-bound quantum ball under the action of attractive two-body and repulsive three-body interactions. There are other suggestions to make a self-bound quantum ball^44,45^. The principal conclusions of the present study should also be applicable to quantum balls prepared in a different fashion. Apart from being of theoretical interest in SSB in nonlinear dynamics, the present study is also of phenomenological interest. To observe the asymmetric Josephson oscillation reported in this paper, one should consider a very weak trap in the *x* and *y* directions so as to localize the quantum ball in an experiment and consider a double-well potential in the *z* direction. Such an experiment seems possible in the future.

## References

[1] Trenkwalder A (2016). Quantum phase transitions with parity-symmetry breaking and hysteresis. Nature Phys..

[2] Zegadlo KB, Dror N, Trippenbach M, Karpierz MA, Malomed BA (2016). Spontaneous symmetry breaking of self-trapped and leaky modes in quasi-double-well potentials. Phys. Rev. A.

[3] Shamriz E, Dror N, Malomed BA (2016). Spontaneous symmetry breaking in a split potential box. Phys. Rev. E.

[4] Mayteevarunyoo T, Malomed BA, Dong G (2008). Spontaneous symmetry breaking in a nonlinear double-well structure. Phys. Rev. A.

[5] Gautam S, Adhikari SK (2015). Spontaneous symmetry breaking in a spin-orbit-coupled f = 2 spinor condensate. Phys. Rev. A.

[6] Adhikari SK (2014). Demixing and symmetry breaking in binary dipolar Bose-Einstein-condensate solitons. Phys. Rev. A.

[7] Cheng Y, Adhikari SK (2010). Symmetry breaking in a localized interacting binary Bose-Einstein condensate in a bichromatic optical lattice. Phys. Rev. A.

[8] Adhikari SK, Malomed BA, Salasnich L, Toigo F (2010). Spontaneous symmetry breaking of Bose-Fermi mixtures in double-well potentials. Phys. Rev. A.

[9] Heil T, Fischer I, Elsässer W, Mulet J, Mirasso CR (2001). Chaos synchronization and spontaneous symmetry-breaking in symmetrically delay-coupled semiconductor lasers. Phys. Rev. Lett..

[10] Hamel P (2015). Spontaneous mirror-symmetry breaking in coupled photonic-crystal nanolasers. Nature Phot..

[11] Malomed BA (2015). Symmetry breaking in laser cavities. Nature Phot..

[12] Sadler LE, Higbie JM, Leslie SR, Vengalattore M, Stamper-Kurn DM (2006). Spontaneous symmetry breaking in a quenched ferromagnetic spinor Bose-Einstein condensate. Nature.

[13] Yukalov VI (2007). Bose-Einstein condensation and gauge symmetry breaking. Laser Phys. Lett..

[14] Scherer M (2013). Spontaneous symmetry breaking in spinor Bose-Einstein condensates. Phys. Rev. A.

[15] Kivshar YS, Malomed BA (1989). Dynamics of solitons in nearly integrable systems. Rev. Mod. Phys..

[16] Salasnich L, Malomed BA (2011). Spontaneous symmetry breaking in linearly coupled disk-shaped Bose-Einstein condensates. Molecular Phys..

[17] Cataliotti FS (2001). Josephson junction arrays with Bose-Einstein condensates. Science.

[18] Levy S, Lahoud E, Shomroni I, Steinhauer J (2007). The a.c. and d.c. Josephson effects in a Bose-Einstein condensate. Nature.

[19] LeBlanc LJ (2011). Dynamics of a Tunable Superfluid Junction. Phys. Rev. Lett..

[20] Adhikari SK (2003). Mean-field model for Josephson oscillation in a Bose-Einstein condensate on an one-dimensional optical trap. Eur. Phys. J. D.

[21] Raghavan S, Smerzi A, Fantoni S, Shenoy SR (1999). Coherent oscillations between two weakly coupled Bose-Einstein condensates: Josephson effects, pi oscillations, and macroscopic quantum self-trapping. Phys. Rev. A.

[22] Xiong B, Gong J-B, Pu H, Bao W-Z, Li B-W (2009). Symmetry breaking and self-trapping of a dipolar Bose-Einstein condensate in a double-well potential. Phys. Rev. A.

[23] Adhikari SK (2014). Self-trapping of a dipolar Bose-Einstein condensate in a double well. Phys. Rev. A.

[24] Ananikian D, Bergeman T (2006). Gross-Pitaevskii equation for Bose particles in a double-well potential: Two-mode models and beyond. Phys. Rev. A.

[25] Adhikari SK, Lu H, Pu H (2009). Self-trapping of a Fermi superfluid in a double-well potential in the Bose-Einstein-condensate-unitarity crossove. Phys. Rev. A.

[26] Adhikari SK (2017). Statics and dynamics of a self-bound matter-wave quantum ball. Phys. Rev. A.

[27] Adhikari SK (2017). Statics and dynamics of a self-bound dipolar matter-wave droplet. Laser Phys. Lett..

[28] Adhikari SK (2016). Elastic collision and molecule formation of spatiotemporal light bullets in a cubic-quintic nonlinear medium. Phys. Rev. E.

[29] Adhikari SK (2017). Elastic collision and breather formation of spatiotemporal vortex light bullets in a cubic-quintic nonlinear medium. Laser Phys. Lett..

[30] Berezhiani VI, Skarka V, Aleksić NB (2001). Dynamics of localized and nonlocalized optical vortex solitons in cubic-quintic nonlinear media. Phys. Rev. E.

[31] Aleksić NB, Skarka V, Timotijevic DV, Gauthier D (2007). Self-stabilized spatiotemporal dynamics of dissipative light bullets generated from inputs without spherical symmetry in three-dimensional Ginzburg-Landau systems. Phys. Rev. A.

[32] Mihalache D (2006). Stable vortex tori in the three-dimensional cubic-quintic Ginzburg-Landau equation. Phys. Rev. Lett..

[33] Perez-Garcia VM, Michinel H, Cirac JI, Lewenstein M, Zoller P (1997). Dynamics of Bose-Einstein condensates: Variational solutions of the Gross-Pitaevskii equations. Phys. Rev. A.

[34] Abraham ERI, McAlexander WI, Sackett CA, Hulet RG (1995). Spectroscopic determination of the S-wave scattering length of lithium. Phys. Rev. Lett..

[35] Shotan Z, Machtey O, Kokkelmans S, Khaykovich L (2014). Three-Body Recombination at Vanishing Scattering Lengths in an Ultracold Bose Gas. Phys. Rev. Lett..

[36] Muruganandam P, Adhikari SK (2009). Fortran programs for the time-dependent Gross-Pitaevskii equation in a fully anisotropic trap. Comput. Phys. Commun..

[37] Vudragović D, Vidanović I, Balaž A, Muruganandam P, Adhikari SK (2012). C programs for solving the time-dependent Gross-Pitaevskii equation in a fully anisotropic trap. Comput. Phys. Commun..

[38] Young-S. LE, Vudragović D, Muruganandam P, Adhikari SK, Balaž A (2016). OpenMP Fortran and C programs for solving the time-dependent Gross-Pitaevskii equation in an anisotropic trap. Comput. Phys. Commun..

[39] Muruganandam P, Adhikari SK (2003). Bose-Einstein condensation dynamics in three dimensions by the pseudospectral and finite-difference methods. J. Phys. B.

[40] Satarić B (2016). Hybrid OpenMP/MPI programs for solving the time-dependent Gross-Pitaevskii equation in a fully anisotropic trap. Comput. Phys. Commun..

[41] Loncar V (2016). CUDA programs for solving the time-dependent dipolar Gross-Pitaevskii equation in an anisotropic trap. Comput. Phys. Commun..

[42] Loncar V (2016). OpenMP, OpenMP/MPI, and CUDA/MPI C programs for solving the time-dependent dipolar Gross-Pitaevskii equation. Comput. Phys. Commun..

[43] Kishor Kumar R (2015). Fortran and C programs for the time-dependent dipolar Gross-Pitaevskii equation in an anisotropic trap. Comput. Phys. Commun..

[44] Maucher F (2011). Rydberg-Induced Solitons: Three-Dimensional Self-Trapping of Matter Waves. Phys. Rev. Lett..

[45] Petrov DS (2015). Quantum mechanical stabilization of a collapsing Bose-Bose mixture. Phys. Rev. Lett..

[46] Young-S., L. E. et al. OpenMP GNU and Intel Fortran programs for solving the time-dependent Gross–Pitaevskii equation. Comput. Phys. Commun. 220, 503-506 (2017).

